# Frequency modulation stimulated Raman scattering scheme for real-time background correction with a single light source

**DOI:** 10.1364/BOE.476513

**Published:** 2022-12-19

**Authors:** Kristin Wallmeier, Thomas Würthwein, Nick Lemberger, Maximilian Brinkmann, Tim Hellwig, Carsten Fallnich

**Affiliations:** 1 University of Münster , Institute of Applied Physics, Corrensstraße 2, 48149 Münster, Germany; 2 Refined Laser Systems GmbH, Mendelstraße 11, 48149 Münster, Germany; 3 University of Münster, Cells in Motion Interfaculty Centre, Münster, Germany

## Abstract

A frequency modulation (FM) scheme for stimulated Raman scattering (SRS) is presented with a single fiber-based light source. Pulse-to-pulse wavelength-switching allows real-time subtraction of parasitic signals leaving only the resonant SRS signal with a noise reduction of up to 30 % compared to digital subtraction schemes, leading effectively to a contrast improvement by a factor of up to 8.3. The wide tuning range of the light source from 1500 cm^−1^ to 3000 cm^−1^ and the possibility to separately adjust the resonant and the nonresonant wavenumber for every specimen allow to investigate a variety of samples with high contrast and high signal-to-noise ratio, e. g., for medical diagnostics.

## 1. Introduction

Stimulated Raman scattering (SRS) is a powerful microscopy technique for in vivo and diagnostic imaging, due to the chemically selective, label-free, and noninvasive nature of the SRS process. Therefore, SRS has been successfully applied in the field of cancer diagnostics [1,2], pharmaceutical research [3] and other medically-relevant fields [4,5]. In contrast to coherent anti-Stokes Raman scattering (CARS), SRS is typically described as being background-free as no intrinsic nonresonant signal is generated by four-wave mixing.

Nevertheless, also in SRS parasitic processes can occur and reduce the contrast within acquired images. For instance two-photon absorption (TPA) reduces the number of detected photons, indicating a loss in the detected beam that can be mistaken as stimulated Raman loss by the simultaneous absorption of (i) two pump photons, (ii) two Stokes photons, or (iii) one pump photon and one Stokes photon [6]. Also processes such as cross-phase modulation (XPM) and thermal lensing can distort the SRS signal. In XPM a high-intensity pump beam changes the refractive index of the sample due to the optical Kerr effect which leads to a modulation of the phase of the probe beam and the generation of frequency-shifted spectral components. The modulation of the refractive index also affects the propagation direction of the light and, therefore, induces a change in the angular distribution of the frequency components of the pulse [7–9]. Hence, some of these frequency components can be truncated by using a too small aperture for the detection and the resulting variation in intensity can be misinterpreted as an SRS signal. Whereas XPM is an instantaneous effect, thermal lensing is a scattering process that appears on a longer time scale as the refractive index changes are induced via temperature fluctuations induced by high-intensity pulses [10] and decay within the thermal relaxation time of the sample in the range of microseconds to milliseconds [11]. The influence of both, XPM and thermal lensing, can be reduced by using a microscope objective with a high numerical aperture (NA) for light collection [12,13]. XPM can be further reduced by using longer pulse durations to decrease the peak power [9], whereas for a further reduction of thermal lensing effects high modulation frequencies in the MHz regime have to be used for averaging out its impact [14]. On the other hand, TPA can be reduced by longer excitation wavelengths in the infrared regime [15]. However, using only the above mentioned techniques is not sufficient to detect a weak SRS signal when a stronger parasitic background signal is present.

Different approaches have already been presented to reduce the parasitic background that appears due to the processes described above, enabling imaging of molecules at low concentrations [16], allowing to visualize structures that are not visible within conventional SRS [12], and making differentiation of constituents within biological samples possible [17]. Stimulated Raman gain and opposite loss detection (SRGOLD) [12] as well as stimulated Raman gain and loss (SRGAL) [13] make use of the symmetric behavior of the parasitic processes and the antisymmetric behavior of the SRS process by detecting the stimulated Raman gain (SRG) and stimulated Raman loss (SRL) simultaneously. When the difference between the separately measured SRL and SRG signals is calculated, the resonant signal is doubled in strength as SRL and SRG have opposite signs, and the parasitic signals cancel due to their symmetric appearance, leading to a two-times increased signal-to-noise ratio (SNR). But the according setups are rather complex with up to three beams at different wavelengths and two separated detection units for acquiring the SRG and the SRL signal. Furthermore, elaborated electronic filtering is required and additional digital image processing, i. e. the pixel-wise subtraction of images, is needed for background correction.

Another approach to suppress the parasitic signals uses frequency modulation (FM) of the excitation pulses. Thereby, a Stokes pulse in combination with pump pulses at two different wavelengths or the other way around are used to probe the resonant SRS signal as well as the parasitic signal at different wavenumbers. By calculating the difference of these two separately measured signals the parasitic background is subtracted, leaving only the resonant signal [18,19]. This subtraction can be done in a digital way by acquiring the resonant signal and the background signal separately one after the other and calculating the difference with a computer [20,21]. Also an analog approach can be used by detecting the alternating resonant and nonresonant signals pixel-wise with a lock-in amplifier [22]. Lock-in detection leads then to a direct background correction before digitization and only one image has to be acquired and digitized instead of two. This allows for noise reduction by a factor of $1-1/\sqrt{2}\approx 30\;\%$ compared to digital post-processing, as for digital calculation the noise of uncorrelated pixels is added in quadrature [23]. For equal signal levels within the analog and the digital approach, this directly leads to an analog advantage of 30 % in the SNR that is given by SNR = ⟨S⟩/σ(S) , with ⟨S⟩ being the average of the signal S and σ(S) being its standard deviation [24,25]. Consequently, as the noise in SRS measurements is proportional to $1/\sqrt{\Delta t}$ with Δt being the integration time, and the SNR is related to Δt by SNR $\propto \sqrt{\Delta t}$ [24], the increase in SNR by 30% allows for faster measurements by roughly 50 %. In the here presented setup the pump pulses are frequency modulated and the SRG on the Stokes pulses is measured, resulting in the same power detected with the photodiode compared to standard SRS, as the pump pulses are filtered out before detection. Therefore, there is not change in the noise contributions as well as the detected signal strength and detected optical power due to the additional power from the second pump wavelength. Taking into account that for the analog FM SRS scheme only one image instead of two images as in the digital FM SRS scheme needs to be acquired, the image acquisition speed is increased by another factor of 2.

Analog as well as digital FM techniques for SRS were implemented in various ways, e. g., by using spectral focusing [16,26]. In spectral focusing two broadband and similarly chirped pulses are used to probe different wavenumbers by changing the temporal delay of the pulses with a mechanical delay line. Within polarization encoding [17], subsequent broadband pulses have orthogonal polarization and are delayed with a birefringent crystal relative to each other to change the probed wavenumber. By using these two techniques the contrast could be increased by a factor of up to 4 and 2, respectively. However, these approaches are limited by a restricted wavelength range, the need for digital post-processing as the resonant and the nonresonant signals could only be acquired separately, or a reduction of the SRS signal due to unavoidable losses in the excitation scheme. Additionally, self-balanced pulse splitting [27] based on self-phase modulation in optical fibers was used for the generation of pulses with different wavelengths to achieve FM SRS in an analog way with limitations regarding the wavelength separation and separate wavelength adjustment of the pulses. For a different approach an angle-to-wavelength pulse shaper [28] was implemented by modulating the incident angle of a beam at a grating and splitting the diffracted and spatially separated frequency components to generate pulses at different wavelengths for FM SRS. This analog scheme allowed for an up to 20-fold reduction of background signal but again just within a limited wavelength range due to the used light source and with a complex setup, where a large part of the available pulse power was wasted. Using a spectrally broadband fs-pulse [29] that was split into separated pulses by multiple wavelength filters, different wavenumbers could be probed by just a single light source, but as before a large part of the pulse power was wasted and although live imaging was possible at different wavenumbers, the background correction had to be done in a digital way by acquiring separate images and subtracting them from each other.

In order to overcome the above mentioned limitations in terms of wavelength range, complexity, and reduced imaging and assessment speed due to digital post-processing, we used a compact as well as fast and widely tunable fiber-based light source providing the necessary pulses for FM SRS and allowing reproducible pulse-to-pulse wavelength-switching for background subtraction via FM SRS within a broad wavenumber range. Additionally, the frequency modulated pump pulses for probing the resonant and parasitic signals at different wavenumbers could be adjusted separately in wavelength and power for optimal wavelength tuning and maximizing background suppression for each sample individually. Due to the alternating probing of the resonant and the nonresonant signal and detection with a lock-in amplifier any digital processing can be avoided and only a single image that is directly background-corrected needs to be acquired. This solution allowed for noise reduction by 30 % and lower pixel dwell time by 50 % compared to the digital approach and enabled live imaging with high contrast, e. g., for the investigation of fast evolving or moving samples.

## 2. Fast and widely tunable fiber-based light source

**Fig. 1.**
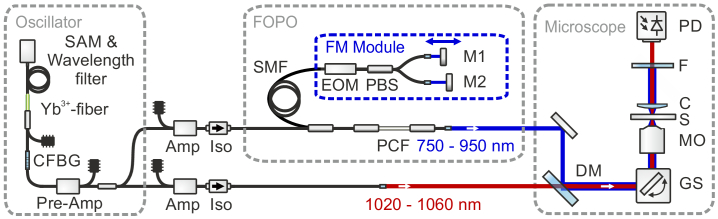
Schematic of the fiber-based light source with an integrated frequency modulation (FM) module for modulating the wavelength of the output of the fiber-based optical parametric oscillator (FOPO) that was synchronously pumped with a mode-locked fiber oscillator. For abbreviations see text.

For the implementation of FM SRS a fiber-based light source (Fig. 1) that was already used for CARS [30,31] and SRS [25] microscopy was modified to provide the wavelength-alternating pump pulses to probe signals at different wavenumbers. The light source consisted of an ytterbium-doped ( ${\mathrm{Yb}}^{3+}$ ) fiber oscillator which was mode-locked with a saturable absorber mirror (SAM). Pulses with a duration of 7 ps, an average power of about 2 mW, and a repetition rate of 40.5 MHz at a central wavelength of 1040 nm were emitted. An electronically tunable filter allowed wavelength tuning between 1020 nm and 1060 nm in less than 5 ms per arbitrary wavelength step [30]. A customized chirped fiber Bragg grating (CFBG) acted as the output coupler of the oscillator and was used to match the dispersion of the oscillator to that of the subsequent fiber-based optical parametric oscillator (FOPO) in order to keep the repetition rates of both cavities of laser and FOPO synchronized for different pump wavelengths, such that a cavity length adjustment could be avoided.

For pumping the FOPO, a part of the oscillator power was amplified (Amp) in two stages to 30 nJ of pulse energy before an isolator (Iso). The linear resonator of the FOPO consisted of about 155 m of polarization-maintaining (PM) single-mode fiber (SMF, Nufern, PM780-HP), 50 cm of PM photonic crystal fiber (PCF, NKT Photonics, LMA-PM-5), a custom-made FM SRS module, and a polished FC/PC connector with a reflectivity of 4 % as the output port, providing a power of approximately 150 mW. Due to the resulting optical resonator length the native repetition rate of the FOPO was 657 kHz. However, the repetition rate of the output pulses was determined by the repetition rate of the oscillator pulses at 40.5 MHz as the FOPO was harmonically pumped, analogous to the effect of harmonic mode-locking [32]. Therefore, multiple pulses with a temporal spacing equal to the spacing of the oscillator pulses were circulating in the FOPO resonator.

The PCF inside the FOPO was used to achieve parametric four-wave mixing (FWM) gain tunable in wavelength between 750 nm and 950 nm by tuning the wavelength of the oscillator pulses. The long fiber resonator enabled spectrally narrow dispersive tuning [30,33], meaning that the pulses inside the FOPO were temporally stretched and only a spectrally narrow part overlapped in time with the subsequent oscillator pulse. This led to a spectral emission bandwidth of less than 12 ${\mathrm{cm}}^{-1}$ over the complete wavelength tuning range, matching the typical vibrational linewidths for SRS [34].

As wavelength-alternating pulses are required for FM SRS an FM module was implemented for pulse-to-pulse wavelength-switching by forming in principle two separated FOPO resonators. The electro-optic modulator (EOM) was synchronized to half of the repetition rate of the oscillator at 20.25 MHz, leading to a corresponding switching in polarization of the FOPO pulses. Accordingly, subsequent pulses could be separated by a polarizing beam splitter (PBS) into two arms and directed to two mirrors (M1, M2), which accomplished two different optical path lengths. The resulting difference in optical cavity length led to different wavelengths at the output due to the dispersive tuning [30,33] in the resonator and, therefore, to stable and reproducible pulse-to-pulse wavelength-switching of the FOPO pulses that acted as pump pulses in the following SRS experiments. For acquiring standard SRS images, one of the arms could be blocked, leading to a 100 % amplitude modulation at 20.25 MHz. In this configuration of the FM SRS module, one of the mirrors was placed on a free-space optical delay line to precisely adjust via its relative position to the fixed mirror the wavelength separation in the FWM gain region. For wavenumbers in the region of the C-D stretching mode around 2125 ${\mathrm{cm}}^{-1}$ for instance, the spontaneous FMW gain ranges approximately from 840 nm to 851 nm. To cover this entire range the mirror needs to be moved by 5.6 cm as shown in Ref. [31]. If pulses with a certain and fixed wavelength difference at the output are acceptable, the free-space optical delay line could be replaced by a completely fiber-integrated mirror.

The other part of the oscillator power was amplified to 400 mW and directly acted as Stokes pulses for the subsequent SRS experiments. In combination with the pump pulses emitted from the FOPO, Raman resonances between 1500 ${\mathrm{cm}}^{-1}$ and 3000 ${\mathrm{cm}}^{-1}$ could be addressed. The beam quality factors were $M^{2}=1.16\pm 0.07$ and $M^{2}=1.03\pm 0.03$ for the Stokes and the pump beam, respectively, making this light source well suited for microscopy applications. In addition, a low noise level of the light source and especially of the unmodulated beam is of high importance for SRS microscopy [35]. Therefore, the relative intensity noise (RIN) of the Stokes beam was determined for characterizing the noise level. The resulting RIN value of -153.7 dBc/Hz for a photodiode current of 3 mA is on a suitable level for SRS imaging as already verified by previous work [25]. Additionally, SRS imaging of tissue sections was successfully shown with a light source providing a 6 dB higher noise level [36], verifying that the achieved noise level of the presented light source is sufficient for SRS microscopy.

## 3. FM SRS imaging

For Raman microscopy the pump and Stokes pulses were overlapped in space by a dichroic mirror (DM) and coupled into a home-built laser-scanning microscope with two nonresonant galvo-scanner mirrors (GS). A 60x microscope objective (MO) with an NA of 0.75 was used to focus into the sample (S) and a condenser lens (C) with an NA of 0.79 collected the light. The stimulated Raman gain on the Stokes beam was acquired with a broadband home-built photodetector (PD), including a large-area photodiode (Hamamatsu, S3590-08) and a low-noise transimpedance amplifier (Texas Instruments, LMH6624), as this detector showed a better contrast performance by a factor of 8 than a commercially available one (APE, SRS Detection Kit). A lock-in amplifier (LIA, Zurich Instruments, HF2LI) measured the signal of the PD and directly provided the background-corrected signal. The image acquisition was controlled by a self-written Matlab program.

Various samples with Raman resonances within a wide wavenumber range that show significant parasitic signals of different origin were investigated to verify the versatile applicability of the FM scheme. All of the following images have 512x512 pixels, were acquired with a LIA time constant of 10 μ s that was the same as the pixel dwell time and are shown without averaging or post-processing. The image artefacts appearing as horizontally mirrored image parts are due to the flyback of the galvo-scanner mirrors.

**Fig. 2.**
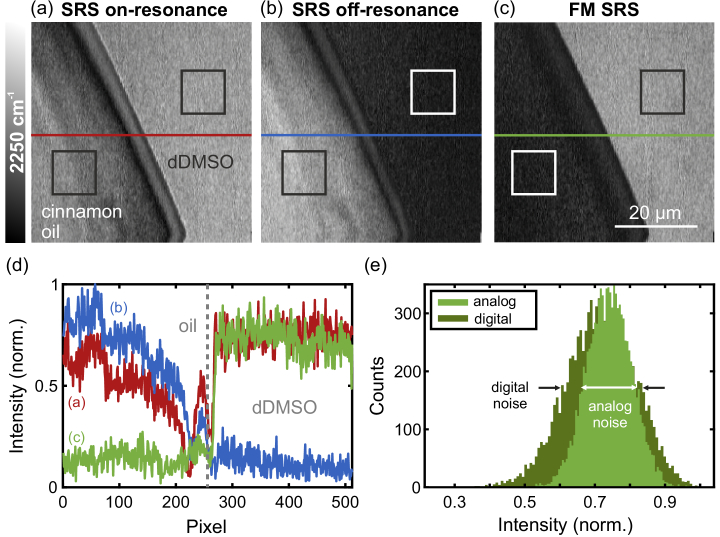
Comparison of standard SRS and FM SRS images of an interface of deuterated dimethyl sulfoxide (dDMSO) and cinnamomum cassia oil at 2250 ${\mathrm{cm}}^{-1}$ . (a) On-resonance image at the resonance of dDMSO, (b) off-resonance image at 2280 ${\mathrm{cm}}^{-1}$ , and (c) FM SRS image with an enhanced contrast by a factor of 3.6. The rectangles show the regions of interest (ROIs) for contrast calculation. All images use the same colormap. (d) Cut through the images along the horizontal lines in (a)-(c). (e) Histogram for the regions of interest (ROIs) within dDMSO for the FM SRS image (c) and a digitally subtracted one (not shown), demonstrating a noise reduction by 30 %.

In Fig. 2(a) an artificial sample consisting of an interface of deuterated dimethyl sulfoxide (dDMSO) and cinnamomum cassia oil is shown, probing the C-D antisymmetric stretching mode of dDMSO at a wavenumber of 2250 ${\mathrm{cm}}^{-1}$ [37] for a pump power of 28 mW and Stokes power of 58 mW in the focal plane. As the two components mix gradually within a few minutes, there is no distinct interface but some region of mixture. Cinnamomum cassia oil consists of aromatic molecules with cinnamyl groups [38]. The included delocalized π -electrons lead to an increased polarizability that in turn leads to a higher nonlinear refractive index by up to an order of magnitude and, therefore, to a strong nonresonant XPM signal [39,40]. The oil with the high nonlinear index of refraction was chosen to demonstrate the effective suppression even of a strong parasitic signal originated by XPM. The nonresonant signal is visible in Fig. 2(a) where the signal of the oil is nearly comparable to the strength of the resonant signal of the dDMSO, although the cinnamomum cassia oil shows no Raman resonances in the region around the probed wavenumber. The effect of XPM is even more pronounced in the off-resonance SRS image in Fig. 2(b), where a wavenumber of roughly 2280 ${\mathrm{cm}}^{-1}$ was probed with the same pump power of 28 mW. Again the oil shows a strong signal, whereas the dDMSO is not resonant for that wavenumber and, therefore, shows less signal compared to Fig. 2(a). The relatively small difference between the probed wavenumbers was chosen to keep the values of light absorption and dispersion in the setup and the sample as much as possible equal at both different wavelengths to ensure also equal intensities. The achieved improvement by using FM SRS is demonstrated in Fig. 2(c) with an increased contrast by a factor of 3.6 compared to standard SRS. The contrast $C=I_{r}/I_{\text{nr}}$ was calculated by taking average values of the marked regions of interest (ROIs) in the images and dividing the according value for the resonant signal of dDMSO ( $I_{r}$ ) by the corresponding one of the nonresonant signal for cinnamomum cassia oil ( $I_{\text{nr}}$ ). In this case the contrast improvement was due to the reduction of the parasitic signal of the oil, as the intensity of the resonant dDMSO signal stayed nearly constant for the standard SRS and the FM SRS image. In Fig. 2(d) a cut along the horizontal lines in the images is shown to demonstrate the suppression of the parasitic signal by leaving the resonant signal essentially unaffected. The red and blue lines represent the intensities for on- and off-resonance SRS images, respectively. For cinnamomum cassia oil, the on- and off-resonance intensities are nearly the same, limited by the different timing of the pump pulses with regard to the Stokes pulse that was optimized for maximum background suppression in the FM SRS image. This does not necessarily mean maximum or minimum signal intensity for the on- and off-resonance images, respectively. In the off-resonance image the signal of dDMSO is clearly reduced compared to the on-resonance image, as there is no significant nonresonant contribution. For FM SRS (green line) the signal of dDMSO is the same as for standard SRS, however, the signal of the oil is reduced, leading to the 3.6-fold contrast improvement. The achieved suppression of parasitic signal verifies that especially for samples with a high nonlinear index of refraction the here presented FM SRS technique can lead to a significant enhancement of contrast, depending on the strengths of the parasitic signal and the resonant signal.

Besides an enhancement of contrast, another advantage of the here presented FM scheme is to provide images with a reduced noise compared to digitally subtracted images, which means the sequential acquisition of two separated images followed by subtraction of each other pixel by pixel with a computer. Here, the noise is defined as the standard deviation of the signal of dDMSO inside the corresponding ROI. For visualizing the achieved noise reduction Fig. 2(e) shows a histogram of the normalized intensity inside the ROI within the dDMSO for the FM SRS image in Fig. 2(c) (light green bins) and a digitally calculated image (dark green bins, equal number of bins for both histograms). The digitally calculated image was computed by subtracting Fig. 2(a) and (b) pixelwise and taking the absolute values to avoid negative intensities. The widths of the histograms are related to the standard deviation and, therefore, to the noise of the signal, which is lower by roughly a factor of 30 % for the analog FM SRS scheme as expected [23]. With this noise reduction while keeping the signal intensity of the resonant signal nearly constant, the SNR is improved by the same factor, enabling a faster image acquisition by up to 50 %.

However, the analog FM SRS method offers an additional advantage over the digital method besides the SNR improvement in that it subtracts off-resonance signal contributions that are not affected by the background signal level of the measurement setup. This advantage results from the different order of the signal subtraction step and the accumulated noise contributions within the data acquisition process in both methods as explained in the following. In the digital approach, small off-resonance signals are hidden in the background noise of the measurement setup because after photodetection, each subsequent signal processing step reduces the SNR due to additive noise, whereas the signal intensity remains constant. Therefore, when subtracting the images in a post-processing step to account for the digital FM SRS signal, a higher value than the intensity of the off-resonance signal is subtracted, resulting in a lower effective FM SRS signal intensity. In case of Fig. 2(e) the reduction is about 4 % compared to the analog scheme. In contrast, in the analog scheme, the FM SRS signal is directly encoded in the pulse modulation and measured accordingly with the photodetector. Therefore, in the analog scheme, small FM SRS signals can still be detected despite the reduction in SNR due to additive noise, resulting in measured intensity values that are corrected by the actual off-resonance signals.

**Fig. 3.**
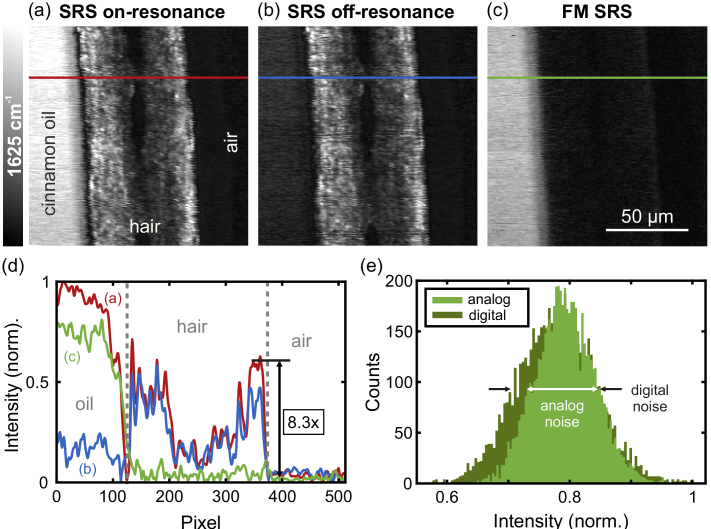
SRS images of a blonde hair placed at the interface of cinnamomum cassia oil and air, probed at the resonance of cinnamomum cassia oil at 1625 ${\mathrm{cm}}^{-1}$ . (a) On-resonance image and (b) off-resonance image with a strong parasitic signal from melanin pigments within the hair. (c) FM SRS image with the parasitic signal suppressed by a factor of 8.3. The cuts along the lines in (a)-(c) are plotted in (d) to demonstrate the suppression. (e) Histogram of the signal of cinnamomum cassia oil around the horizontal line for a digitally subtracted image and the corresponding analog FM SRS image (c) with 30 % lower noise.

For presenting the adaptability of the FM SRS scheme also for different wavenumbers without further readjustment and within 5 ms of switching time as well as the applicability not only for an artificial sample but also for biological samples a blonde hair at an interface of cinnamomum cassia oil and air was investigated. The blonde hair was chosen as the melanin pigments inside the hair are known to show a strong parasitic signal induced by TPA [28]. The on-resonance SRS image probed at the resonance of the oil at 1625 ${\mathrm{cm}}^{-1}$ [41] (Fig. 3(a)) shows a strong resonant signal from the cinnamomum cassia oil as well as a strong signal arising from melanin pigments in the outer regions of the hair. The power was set to 23 mW for the Stokes and 12 mW for the pump beam for the measurement. The off-resonance image in Fig. 3(b) at a wavenumber approximately 30 ${\mathrm{cm}}^{-1}$ above the resonance of the oil verifies that the melanin led to a nonresonant parasitic signal, induced by TPA, thus being independent of the probed wavenumber. In the FM SRS image (Fig. 3(c)) this parasitic signal is successfully suppressed by a factor of 8.3 down to the level of background noise, giving access to the resonant signal of cinnamomum cassia oil only. The intensities along the horizontal lines in the images are shown in Fig. 3(d) to visualize the reduction of the TPA signal. The intensity of the oil is slightly decreased within the FM SRS image compared to the on-resonance image as the oil again shows a nonresonant signal in addition to the resonant signal that is subtracted by the FM scheme. The parasitic signal from melanin decreases to the level of background noise that was at the same signal level as from air within the sample as being a reference with no SRS signal at the used power levels, verifying the significant suppression of the parasitic signal. The factor of 8.3 was calculated by determining the ratio of the average values inside ROIs along the horizontal lines in the outer region of the hair for the on-resonance SRS and the FM SRS image as indicated exemplary in Fig. 3(d).

As before, the noise of the signal of the oil, more specifically the standard deviation, was determined to be lower by roughly 30 % for the FM SRS image compared to the digitally subtracted image, leading to a higher SNR and underlining the advantage of the here used analog FM scheme in contrast to a digital approach. For comparison the histograms of the analogously and digitally subtracted images are shown in Fig. 3(e), wherein ROIs along the horizontal line in the part of the image with the resonant signal, i. e. the part with the cinnamomum cassia oil, were used to calculate the standard deviation and to generate the histograms.

In order to demonstrate the real-time capability of FM SRS imaging a video (4 frames per second, LIA time constant 1 μ s) is attached to the supplementary material (Visualization 1), showing live background correction for the above investigated sample consisting of cinnamomum cassia oil, air, and a blonde hair at 1625 ${\mathrm{cm}}^{-1}$ . Additionally, fast switching between standard on- and off-resonance SRS imaging as well as FM SRS imaging is shown by simply (un-)blocking one of the feedback arms in the FOPO.

**Fig. 4.**
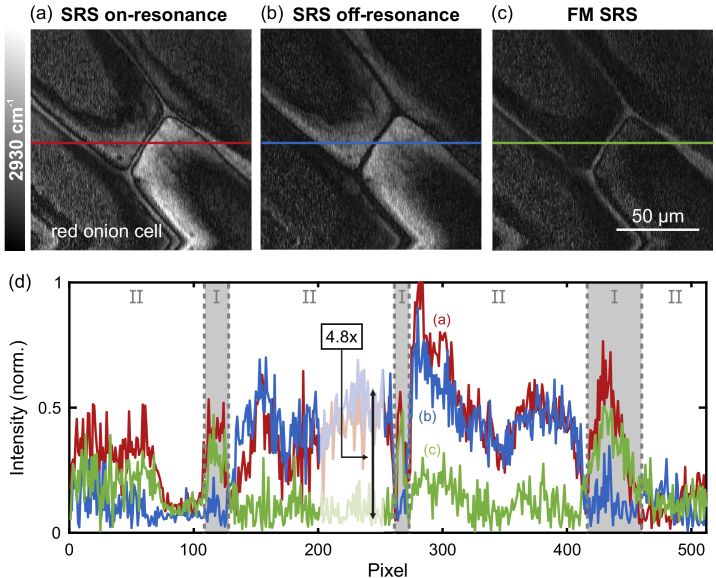
Comparison of standard SRS and FM SRS images of red onion cells probed at 2930 ${\mathrm{cm}}^{-1}$ . (a) On-resonance and (b) off-resonance SRS image with a strong parasitic signal arising from two-photon absorption of pigments within the cells. (c) FM SRS image with 4.8-fold suppression of the parasitic signal. (d) Cut along the horizontal lines to demonstrate the signal intensities within the cell walls (I) and the inner side of the cells (II).

Due to the wide wavelength tuning of the light source in combination with the FM scheme, also samples in the CH-stretch region could be investigated with high contrast, which is of interest for medical applications, e. g., for cancer diagnostics [1,2]. In order to demonstrate the contrast improvement for biological imaging, red onion cells were investigated at the ${\mathrm{CH}}_{3}$ -resonance at 2930 ${\mathrm{cm}}^{-1}$ [42] (see Fig. 4(a)) with power levels of 59 mW and 20 mW for Stokes and pump beam, respectively. The red pigments inside the cells showed a strong nonresonant TPA signal [43] that was even stronger than the resonant signal of the cell walls (Fig. 4(b), wavenumber was set to 2880 ${\mathrm{cm}}^{-1}$ ), whereby it was challenging to get only the desired information from the resonant signal of the sample. However, by using FM SRS (Fig. 4(c)) the parasitic signal could be removed, giving access only to the resonant SRS signal. Again intensity profiles along the horizontal lines are shown for comparison in Fig. 4(d) to demonstrate the significant suppression of the nonresonant signal. The resonant signal, that is generated within the cell walls (section 1), was nearly the same for standard SRS and FM SRS, whereas the signal strength from the inner side of the cells (section 2) was clearly reduced by up to a factor of 4.8 as indicated exemplary within the line plot, enabling the investigation of only the relevant parts of the sample that show a Raman resonance at the adjusted wavenumber.

## 4. Conclusion

We demonstrated a light source for stimulated Raman scattering (SRS) with an integrated frequency modulation (FM) module, such that only a single light source was needed for pulse generation at three different wavelengths, enabling live background correction by FM SRS. The light source was fast and widely tunable in wavelength, allowing to probe Raman resonances from 1500 ${\mathrm{cm}}^{-1}$ to 3000 ${\mathrm{cm}}^{-1}$ . The pulse-to-pulse wavelength-switching mechanism allowed for modulation at half of the repetition rate, enabling the imaging of rapidly evolving samples, actually only limited by the scanning time in the range of a few hundred milliseconds per image. Additionally, changing the setup for acquiring standard SRS images or FM SRS images was achieved just by blocking or unblocking one of the feedback arms of the fiber-based optical parametric oscillator, respectively. Although SRS is typically denoted in the scientific community as being a background-free coherent Raman scattering process, suppression of parasitic signals by up to a factor of 8.3 down to the level of background noise was achieved for artificial as well as biological samples at different wavenumbers within a broad wavenumber range in real-time without the need for readjustment, verifying the applicability of the technique for medical or biological research with high contrast. Additionally, the residual noise level of the images was reduced compared to digital subtraction schemes by up to the theoretically maximum achievable value of 30 %, leading to a higher signal-to-noise ratio by up to 30 % and enabling reduced pixel dwell time by up to 50 %. In combination with the robustness of the light source the presented FM scheme offers the potential for microscopy applications in medical diagnostics or environmental sensing with enhanced contrast even in challenging surroundings.

## Data Availability

Data underlying the results presented in this paper are not publicly available at this time but may be obtained from the authors upon reasonable request.
